# Dynamic Programming Used to Align Protein Structures with a Spectrum Is Robust

**DOI:** 10.3390/biology2041296

**Published:** 2013-11-20

**Authors:** Allen Holder, Jacqueline Simon, Jonathon Strauser, Jonathan Taylor, Yosi Shibberu

**Affiliations:** 1 Department of Mathematics, Rose-Hulman Institute of Technology, Terre Haute, IN 47803, USA; E-Mails: simonjc@rose-hulman.edu (J.S.); taylorj7@rose-hulman.edu (J.T.); shibberu@rose-hulman.edu (Y.S.); 2 Roche Diagnostics, Indianapolis, IN 46256, USA; E-Mail: strauserj@upmc.edu

**Keywords:** protein alignment, structural bioinformatics, dynamic programming

## Abstract

Several efficient algorithms to conduct pairwise comparisons among large databases of protein structures have emerged in the recent literature. The central theme is the design of a measure between the *C_α_* atoms of two protein chains, from which dynamic programming is used to compute an alignment. The efficiency and efficacy of these algorithms allows large-scale computational studies that would have been previously impractical. The computational study herein shows that the structural alignment algorithm eigen-decomposition alignment with the spectrum (EIGAs) is robust against both parametric and structural variation.

## 1. Introduction

The problem of aligning protein structures to infer functional similarity is a stalwart within the arena of computational biology; see [1,2,3,4,5,6,7,8] as recent examples. Over the last couple of years, there have been several publications that have promoted fast algorithms for database-wide comparisons [1,8,9,10] (see the related work in [11,12,13,14,15,16,17,18,19]). These algorithms are designed to efficiently discern if two proteins share a similar molecular structure. Since structural similarity often aligns with functional similarity, these algorithms promote making pairwise comparisons among large databases with the intent being to identify functionally similar groups.

Classical structure alignment algorithms like Dali [20], CE [21], TM-align [22] and SSAP [23] provide accurate alignments, but are typically too slow for efficient all-against-all structural comparisons or database searches. One method of accelerating an algorithm is to modify it to run on a GPU (graphics card). Pang *et al* [24] report a 36-fold speedup for TM-align modified for GPUs. Another approach is to reduce the search space by pruning unlikely matches through a process that clusters structures and then compares cluster representatives [25]. However, such comparisons are typically between structures with low similarity, and the work of Pascual-Garcia *et al.* [26] provides evidence that low structural similarity comparisons are better represented by a network of similarities rather than discrete fold clusters. The issue is that low structural similarity can lead to the loss of transitivity, *i.e.*, protein A may be similar to protein B and protein B similar to protein C, and yet A may not be similar to C. A third approach is to combine a fast algorithm to filter comparisons with a slower algorithm to increase accuracy. For example, CATHEDRAL [27] combines a fast secondary structure-based algorithm with a slower double-dynamic programming algorithm.

The modern cohort of fast structural alignment algorithms sacrifices accuracy with regard to the pairwise comparisons in exchange for increased speed. Many of the fast algorithms (so-called 1D algorithms [3]) have a single application of dynamic programming (DP) at their core. However, so called 0D, or “finger-print,” algorithms [3] have emerged that are, in principle, even faster than DP-based 1D algorithms. FragBag [28] is one such 0D algorithm. FragBag represents protein structures as histograms of backbone fragments, and its computational speed is better than DP, due to the efficiency of comparing histograms.

The speed with which the most recent algorithms can compare proteins prompts a wealth of numerical studies that would have been previously difficult, if not impossible. We harness this efficiency to test if the structural alignment algorithm eigen-decomposition with the spectrum (EIGAs) is robust with regards to parametric variation and modeling uncertainty. Like many of the recent, efficient algorithms, EIGAs uses dynamic programming (DP) as its underlying computational framework. DP depends on parameters that generally need to be tuned to the application and, in some cases, to the particular problem instance. This begs the question of how sensitive the recent DP-based algorithms are to selecting quality parameters. If parameters have to be selected somewhat precisely to obtain accuracy and/or have to be tuned per dataset, then the technique would lack sufficient robustness to instill confidence on much larger datasets, which would preclude individualized parametric tuning. We show that EIGAs is robust to parameter selection by showing that it remains highly effective at identifying structurally similar proteins over a breadth of parametric values.

The optimization model solved by DP depends on a measure of the structural similarity between two proteins. However, the coordinates of a residue are not known with perfect certainty, and the structural similarity measure and, subsequently, the alignments themselves are subject to possible adjustments, as a protein's 3D structural description varies within appropriate tolerances. As with parametric variation, if an algorithm is sensitive to small variations in structure, then it will likely perform poorly, since real datasets always have a level of uncertainty. We show that EIGAs is robust against such uncertainty by showing that it identifies a preponderance of structurally similar proteins as the coordinates of the *C_α_* atoms are perturbed randomly. We account for uncertainty by assuming that the atomic coordinates of the *C_α_* atoms are probability distributions, whose standard deviations are scaled B-factors.

The next section discusses how DP is used to efficiently align protein structures. This discussion points to why DP might have a natural affinity for accounting for parametric variation and structural uncertainty. Sections 3 and 4 specify EIGAs' DP model and detail the computational environment used to conduct our numerical experiments. Section 5 reports on our numerical tests showing that EIGAs is robust against parametric adjustment. Section 6 reports on the robustness of EIGAs as coordinate uncertainty is considered.

## 2. Dynamic Programming and Robustness

Dynamic programming originated with Bellman [29] and was first used to align biological sequences by Needleman and Wunsch [30]. The discrete setting of structural alignment means that the iterative results of the DP recursion are decided by selecting a best value from among a few possibilities. This immediately points to the fact that these numbers can adjust over nearby values without altering the optimal solution, and it is this observation that we promote.

Suppose we want to align two sequences, one indexed by *i* and the other by *j*. Allowing *S_ij_*, to be the similarity between element *i* of the first sequence and element *j* of the second, we have that a common recursion defining an optimal alignment is:

(1) $$V_{ij}=\min\;\{\begin{array}{l} V_{i-1,j}+\rho \\ V_{i,j-1}+\rho \\ V_{i-1,j-1}+S_{ij}, \end{array}$$

where *ρ* is a penalty for inserting or continuing a gap. Calculating the recursion over *i* and *j* produces the optimal value over all possible alignments, and an optimal alignment itself can be calculated by backtracing the steps used to construct the optimal value. As an illustrative example, assume the similarity values are tabulated so that:

$$\begin{array}{c} \begin{array}{cccccc} & \;j=1 & j=2 & j=3 & j=4 & j=5 \end{array}\\ \begin{array}{cc} \begin{array}{c} i=1\\ i=2\\ i=3\\ i=4 \end{array} & \left[\begin{array}{ccccc} 0.1 & \;0.2 & \;0.8 & \;0.7 & \;1.2\\ 0.5 & \;0.4 & \;0.2 & \;0.1 & \;0.5\\ 0.2 & \;0.3 & \;0.1 & \;0.2 & \;0.2\\ 0.8 & \;0.7 & \;0.6 & \;0.1 & \;0.1 \end{array}\right] \end{array} \end{array}=S.$$

The values of *V* with *ρ* = 0.3 are calculated from the recursion in (1):

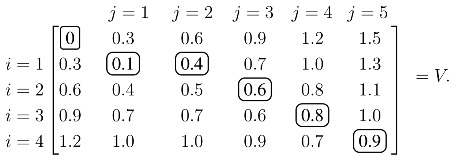

Each element of *V*, except the initial zero in the upper left, is calculated from (1). For example, the 0.4 in the *i* = 1, *j* = 2 position is:

$$0.4=\min\;\{\begin{array}{ll} 0.1+0.3 & \left(\text{add gap penalty from left}\right)\\ 0.3+0.2 & \left(\text{pair}\;i=1\;\text{with}\;j=2\;\text{from the upper left dignoal}\right)\\ 0.6+0.3 & \left(\text{add gap penalty from top}\right) \end{array}$$

Once *V* is constructed, the score of the optimal alignment is in the bottom right element, and the alignment itself is calculated by backtracing a path that produced this score. In this case, the unique elements of the optimal path are circled, and the optimal alignment/solution is:
Protein A1_234Protein B12345
Pair Value0.10.30.20.20.1.

An optimal solution depends on the gap penalty, *ρ*, and the individual values of the structural similarity measure, *S_ij_*. However, solutions are not necessarily sensitive to changes in these values. Indeed, a simple, albeit tedious, calculation shows that the example's unique solution remains optimal over the substantial range of 0.15 < *ρ* < ∞. Similar calculations show that each individual, *S_ij_*, may vary with *ρ* = 0.3 over the intervals noted below:

$$\left[\begin{array}{lllll} \left(0.0,0.2\right) & \left(0.1,\infty \right) & \left(0.0,\infty \right) & \left(0.0,\infty \right) & \left(0.0,\infty \right)\\ \left(0.0,\infty \right) & \left(0.3,\infty \right) & \left(0.0,0.3\right) & \left(0.0,\infty \right) & \left(0.0,\infty \right)\\ \left(0.0,\infty \right) & \left(0.0,\infty \right) & \left(0.0,\infty \right) & \left(0.0,0.3\right) & \left(0.0,\infty \right)\\ \left(0.0,\infty \right) & \left(0.0,\infty \right) & \left(0.0,\infty \right) & \left(0.0,\infty \right) & \left(0.0,0.2\right) \end{array}\right].$$

This example illustrates that a solution from DP is stable as the input data is slightly, and, in many cases, robustly, perturbed. The sizes of the stability ranges depend on the particular problem, and they can be quite small. However, changes in the alignment resulting from a slight extension beyond one of these intervals can be minor, and the resulting score may still suffice as a delineating value. The fundamental question of this paper is whether or not the efficacy of EIGAs degrades as its input data is adjusted. If so, then either it is highly sensitive to changes in its algorithmic parameters, such as the gap penalty, *ρ*, or it is highly sensitive to minor adjustments in its model of structural similarity, *S*.

There are numerous variants of the simple recursion in (1). The gap penalty, *ρ*, in the example penalizes opening and continuing a gap identically. If the penalties for opening and continuing a gap are different, then an affine gap penalty is being used. Some studies in computational biology have indicated a benefit to an affine gap model [31,32]. However, affine models require an additional parameter over their non-affine counterparts, which complicates parameter tuning for the application at hand. Our numerical work uses an affine gap penalty, and we let *ρ_o_* and *ρ_c_* be the penalties for initiating and continuing a gap, respectively.

While the discussion above depicts DP favorably, we would be remiss to ignore some of its downsides. All uses of DP for database applications only consider sequential alignments. Non-sequential alignments are important in some, and, indeed, possibly many, cases [33,34,35]. Adapting DP to accommodate for non-sequential alignments, say by re-ordering the manner in which DP iterates along the backbone, would be an important direction for future research. The use of bipartite graph matching in place of DP to obtain non-sequential alignments should also be investigated [36].

## 3. Eigen-Decomposition with the Spectrum

A protein (chain) is a linear polymer of amino acid residues that folds into a unique 3D structure. The amino acid residues are held together with strong covalent bonds, and the covalent backbone is comprised of a repeating pattern of atoms identical for each residue. However, each residue is distinguished by a side chain of atoms that is not on the backbone, but is attached to the alpha carbon backbone atom, denoted *C_α_*, for each residue. We represent each residue by the coordinates of its *C_α_* atom. An alignment between two protein chains is a pairing of residues between the proteins, which reduces to a pairing between the *C_α_* atoms along the two backbones.

Let *d_kt_* be the distance between residues *k* and *t* of a single protein. The smooth contact matrix for the protein is:

$$C_{kt}=\{\begin{array}{cc} 1-d_{kt}/k, & \text{if}\;d_{ij}\leq k\\ 0, & \text{otherwise}. \end{array}$$

The parameter, *κ*, defines the largest distance at which the model permits a nonzero relationship between residues *k* and *t*. Residue pairs whose distance is less than *κ* receive a linearly-scaled value of *d_kt_*. We note that *C_kk_* = 1, since *d_kk_* = 0. This property implies that *C* is positive definite for appropriately selected *κ* [37], and hence, *C* can be factored as *C* = *UDU^T^*, where *U^T^* = *U*^−1^ and *D* is a diagonal matrix of positive eigenvalues. If we let $R=\sqrt{D}U^{T}$, then *C* = *R^T^ R*. The column vectors of *R* are called intrinsic contact vectors, and they support a geometric perspective of the alignment problem [8].

The similarity between residues *i* and *j* of different proteins is *S_ij_* = |λ*_i_* − λ_j_|/|λ*_i_* + λ*_j_*|, where λ*_i_* and λ*_j_* are the eigenvalues associated with the two residues. Specifically, residue *i* is assigned λ*_p_* if |*R_pi_*| = max*_q_* |*R_qi_*|; see [8] for additional details. This eigenvalue assignment is not that of principle component analysis (PCA) or normal mode analysis (NMA), as it includes many smaller eigenvalues, which are typically more sensitive to deviations in the input data. As an example, the distribution of EIGAs' eigenvalue assignments for the Proteous300 dataset is tabulated below.


smallestlargest
size10%20%30%40%50%60%70%80%90%100%assignments0.42%4.03%6.36%9.15%11.41%18.06%12.18%9.66%3.93%24.79%

Nearly 75% of the residues are assigned eigenvalues whose magnitude is below 90% of the largest eigenvalue, and about 31% of the residues are assigned eigenvalues whose magnitude is below 50% of the largest eigenvalue. Unlike PCA or NMA, EIGAs' assignment associates each residue with the eigenvalue of the nearest eigenspace, and as the distribution above shows, many of a protein's more minor eigenvalues are used by EIGAs.

## 4. The Computational Setting

The EIGAs code is implemented in Python, although portions, such as the DP core, are written in C as Python extensions via SWIG [38]. BioPython [39] is used to parse the protein database (pdb) files, and NumPy is used to factor the smooth contact matrices. All computations for this study were completed on a 2.2 GHz chipset with 7 G of RAM, and all tests were conducted on the Proteus300 [1] dataset, which consists of 300 proteins belonging to 30 known families, as identified by the Structural Classification of Proteins (SCOP) database [40,41,42]. EIGAs requires about three minutes to complete the 44,850 pairwise comparisons of Proteus300 in serial (about 0.0041 seconds per comparison). The preprocessing step of encoding each of the 300 proteins as a list of eigenvalues also requires about three minutes (approximately 0.6240 seconds per protein). Using Python's multiprocessing package, the same task can be distributed to different CPUs, although we have not experienced speed-ups.

EIGAs' computational efficiency compares favorably with other published results. The Eig_7 algorithm in [12] can complete the Proteus300 dataset in about six hours, and A_purva [1] is able to finish the dataset in about 13.5 hours. The numerical results in [9] show that GOSSIP can complete the Proteus300 dataset in as little as 20 minutes and 44 seconds. Kpax [43] reports that it can accomplish 2,980 comparisons per second, which equates to about 15 seconds for Proteus300. LNA has the fastest reported results on GPUs and is capable of finishing the Proteus300 dataset in under a second [9].

The earlier numerical results of EIGAs in [8] have been improved in many ways. The earlier version of EIGAs used only a non-affine gap model, and the DP algorithm had been coded in Matlab. We gained a significant speedup with a C implementation of DP. EIGAs had previously used a non-affine gap penalty of one along with the un-normalized similarity value of *S_ij_* = |λ*_i_* − λ*_j_*|. EIGAs now uses an affine gap model, and similarity values are normalized as *S_ij_* = |λ*_i_* − λ*_j_*|/|λ*_i_* + λ*_j_*|, which ensures that the score is always between zero and one. Lastly, the previous benchmark had been a simple count of the proteins whose nearest neighbor had shared the same family classification. Assessments are now made from a receiver operating characteristic (ROC) curve, which is an improved evaluative tool that parameterizes the trade-off between positive and negative results.

## 5. Robustness Against Parametric Variation

Three algorithmic parameters affect alignments, those being the cutoff value, *κ*, the gap opening penalty, *ρ_o_*, and the gap continuation penalty, *ρ_c_*. We incremented *κ* from four to 23 Å with a step size of 1 Å and incremented each of the gap penalties from zero to one with a step size of 0.1. The 30 families of the Proteus300 dataset were randomly divided into three sub-groups of 10 families (100 proteins) each. We adapted a standard three-fold cross-validation test in an attempt to quantify how parametric tuning on a small collection of proteins might foreshadow success on a larger, and possibly disjoint, dataset. Instead of tuning on two of the three sub-groups and then validating on the third, parameters were tuned on a single dataset and then validated on the remaining two. The tuning on each sub-group was over the space of all 20 × 10 × 10 = 2,000 triples (*κ*,*ρ_o_*,*ρ_c_*). Hence, each of the three tunings required $2,000\times \left(\begin{array}{c} 100\\ 2 \end{array}\right)=9,900,000$ applications of DP, which, at 0.0041 seconds per solve, is about 11.5 hours.

An ROC curve is generated for each (*κ*, *ρ_o_*, *ρ_c_*), which is a parametric plot of the false positive rate (FPR) against the true positive rate (TPR), as the value deciding structural similarity varies. As an example, suppose the optimal alignments among five proteins have the optimal DP values in Table 1. The best (minimum) agreement is between proteins C and E, which are aligned by EIGAs' DP algorithm with a globally optimal value of 0.08. The worst agreement is between proteins A and E, which even when optimally aligned by EIGAs receive a score of 0.68. Let *τ* satisfy 0.08 ≤ *τ* ≤ 0.68, and for the moment, consider *τ* = 0.20. Only three alignments have a value below *τ*, those being between pairs (A,D), (C,E) and (D,E), but only the first two correctly pair proteins within the same family. There are a total of 10 possible pairs in this example, but only four correctly pair proteins of the same family. Therefore, for *τ* = 0.20, the TPR is 2/4, *i.e.*, we correctly identified two of the four pairings that share the same family. Since (D,E) was the only false positive out of six possible, the FPR for *τ* = 0.20 is 1/6. Varying *τ* over the interval [0.08, 0.68] gives a parametric plot of FPR *versus* TPR. Note that TPR and FPR are both one for *τ* ≤ 0.08 and are both zero for *τ* > 0.68.

**Table 1:** Illustrative values assigned to optimal alignments from dynamic programming (DP).

	Prot.A	Prot. B	Prot. C	Prot. D	Prot. E
Prot. A	0.00	0.21	0.43	0.13	0.68
Prot. B	0.21	0.00	0.61	0.26	0.34
Prot. C	0.43	0.61	0.00	0.80	0.08
Prot. D	0.13	0.26	0.80	0.00	0.19
Prot. E	0.68	0.34	0.08	0.19	0.00

Family ID	1	1	2	1	2

A perfect classification has a TPR of one and an FPR of zero, and hence, it is desirable to have a ROC curve pass as close to this point as possible. A random classifier gives identical TPR and FPR values. Therefore, if an ROC curve is above the TPR = FPR line, then the classification method is better than random classification, but if the ROC curve is below the TPR = FPR line, then the classification is worse than random. A common metric is the area under the ROC curve (AUROC), which is at best one and at worst zero. The area of the ROC curve along with its minimum distance to a perfect classification are the metrics used to evaluate the alignments for each (*κ*, *ρ_o_*, * ρ_c_*).

**Figure 1:**
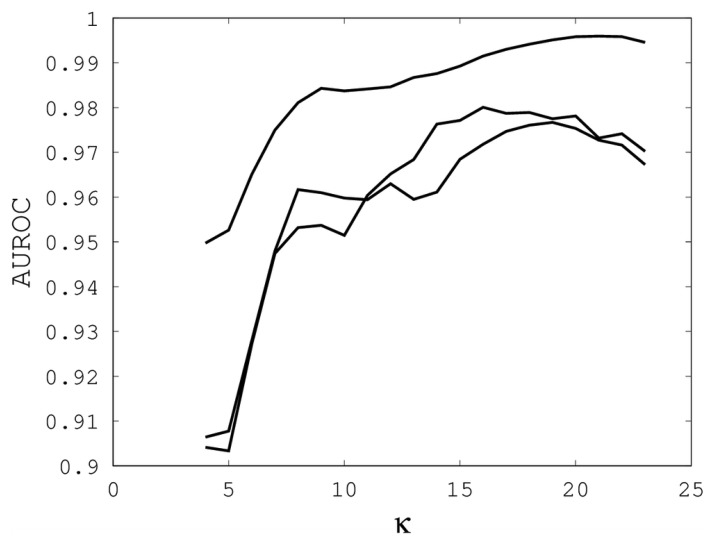
The best area under the receiver operating characteristic curve (AUROC) over all (*ρ_o_*, *ρ_c_*) for each *k*. Each of the three curves corresponds with one of the random sub-groups of the Proteus300 dataset.

**Figure 2:**
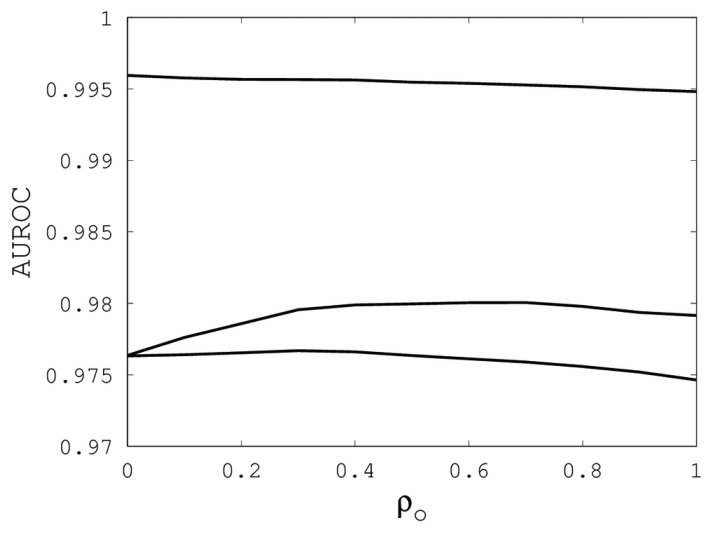
The best AUROC over all (*k*, *ρ_c_*) for each *ρ_o_*. Each of the three curves corresponds with one of the random sub-groups of the Proteus300 dataset.

**Figure 3:**
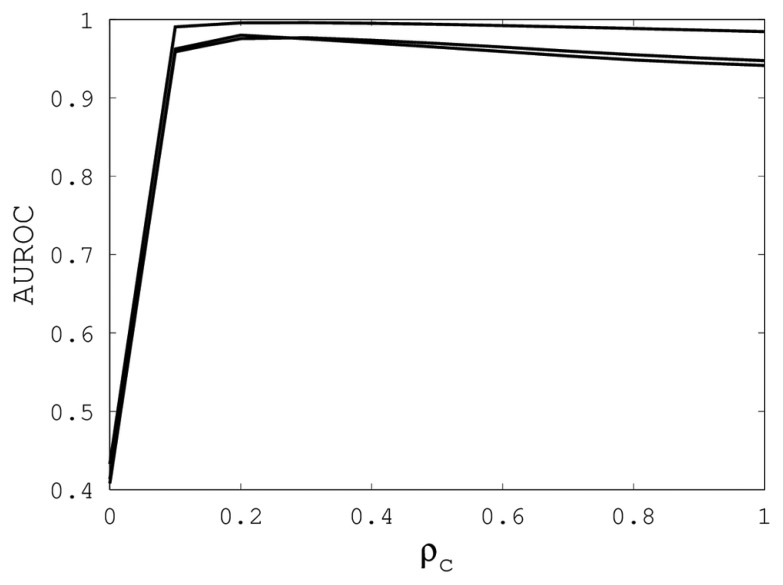
The best AUROC over all (*κ*, *ρ_o_*) for each *ρ_c_*. Each of the three curves corresponds with one of the random sub-groups of the Proteus300 dataset.

Figures 1, 2 and 3 illustrate the sensitivity of EIGAs' alignments as evaluated by AUROC. For each individual value of *κ*, *ρ_o_* and *ρ_c_*, the best possible AUROC over the other two parameters is plotted. The sensitivity is greatest with respect to *κ*, and while AUROC exceeded 0.9 in all cases, the sharp rise to well over 0.95 as *κ* increases demonstrates that *κ* should not be small. The best values were *κ* = 19, 21, and 16 over the three sub-groups for respective best AUROCs of 0.9767, 0.9959 and 0.9801. The alignments were nearly insensitive to *ρ_o_*, as illustrated by the nearly constant graphs in Figure 2. Indeed, each *ρ_o_* yielded an AUROC exceeding 0.97. From Figure 3, we see that *ρ_c_* should not be selected too small, since, if so, the alignments do not accurately predict family classification. However, quality alignments with AUROCs over 0.95 are possible, as long as 0.1 ≤ *ρ_c_* ≤ 0.9. The best values of *ρ_c_* over the three sub-groups are 0.3, 0.3 and 0.2 with corresponding AUROCs of 0.9767, 0.9960 and 0.9801.

Our computational results show that AUROC exceed 0.95, as long as 7 ≤ *κ* ≤ 23, 0 ≤ *ρ_o_* ≤ 1 and 0.1 ≤ *ρ_c_* ≤ 0.9. These parametric ranges are wide and account for over 67% of the tested parameters. We conclude that EIGAs is robust with respect to parametric tuning. This outcome suggests that it might be possible to tune parameters for general use on small datasets. To test this concept, we used the parameters with the best AUROC from each sub-group on the remaining proteins of Proteus300. The results are shown in Table 2, and they demonstrate that it is reasonable to assume that parameter tuning on smaller datasets will result in quality parameters for larger datasets. On average, AUROC degraded from 0.9842 to 0.9742 over the three sub-group tests, although AUROC increased on the first sub-group. The TPR and FPR values listed in Table 2 are those closest to perfect classification, and they show that 90% of EIGAs' alignments correctly pair proteins of the same family, assuming that an appropriate threshold, *τ*, is selected. Likewise, less than 9% of family associations predicted by EIGAs are incorrect.

**Figure 4:**
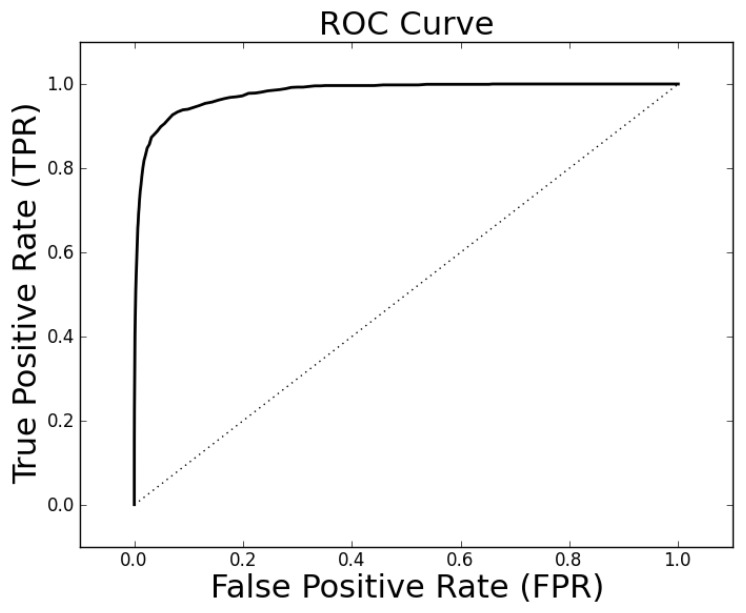
The receiver operating characteristic (ROC) curve for the entire Proteus300 dataset with *κ* = 19, *p_o_* = 0.5 and *p_c_* = 0.3.

The parameter tuning suggests that *k* = 19, *ρ_o_* = 0.5 and *ρ_c_* = 0.3 will generally result in alignments of sufficient quality to identify a high percentage of proteins with the same family classification. Using these parameters on the entire Proteus300 dataset gives an AUROC of 0.9787 and a best TPR and FPR of 0.9267 and 0.0704. The ROC curve is shown in Figure 4. As a point of observation, the EIGAs scoring method consistently resulted in the best TPR and FPR for 0.12 ≤ *τ* ≤ 0.15. The best TPR and FPR for the entire Proteus300 dataset occurred at *τ* = 0.1458.

**Table 2:** Parameters *κ*, *ρ_o_* and *ρ_c_* were tuned to give the best possible AUROC for three randomly selected sub-groups of Proteus300. The results of using these parameters on the remaining, disjoint set of proteins are listed in the last three columns. TPR, true positive rate; FPR, false positive rate.

	Best Tuned Parameters per Sub-Group				Results on Remaining Proteins		

	AUROC	*κ*	*ρ_o_*	*ρ_c_*	AUROC	TPR	FPR

Sub-Group 1	0.9767	19	0.3	0.3	0.9801	0.9389	0.0724
Sub-Group 2	0.9959	21	0.0	0.3	0.9696	0.9067	0.0836
Sub-Group 3	0.9801	16	0.7	0.2	0.9728	0.9100	0.0848

## 6. Robustness Against Coordinate Uncertainty

Beyond parametric variation, optimal alignments also depend on the *C_α_* coordinates, since they decide the similarity values, *S_ij_*, for a fixed value of *κ*. As far as we are aware, no computational evaluation has been proposed to measure an algorithm's ability to account for 3D variability, which is not surprising, since earlier alignment algorithms required lengthy computations. However, EIGAs' speed supports the repeated database-wide comparisons needed to study how it reacts as protein descriptions vary over experimental tolerances.

There are two common methods for establishing a protein's structure: X-ray crystallography and nuclear magnetic resonance (NMR) spectroscopy. Each accounts for variability differently, and these differences need to be reconciled to have a consistent study of coordinate uncertainty. We reconcile the differences probabilistically and assume that the coordinates of each *C_α_* are random variables that can be modeled from either experiment. We then draw a sample dataset from these distributions and align them with EIGAs. Each run of EIGAs on a sample dataset is evaluated by AUROC, with *κ* = 19, *ρ_o_* = 0.5 and *ρ_c_* = 0.3, as determined in Section 5.

**Figure 5:**
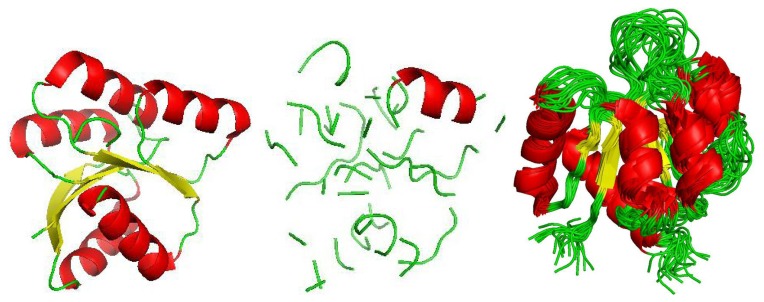
An unperturbed depiction of 1QMPa is shown on the left, and a sample from a random model of the same protein chain is shown in the center (*s* = 1). The image on the right is an overlay of all nuclear magnetic resonance (NMR) renderings of 1NTR.

NMR experiments generate multiple descriptions of a protein in aqueous solution, and pdb files from NMR experiments include numerous 3D models. An overlay of all 20 models from the NMR experiment of the protein structure 1NTR is shown in Figure 5. The NMR technique is advantageous because proteins are in a natural, aqueous environment, and hence, the renderings hopefully represent conformations of a folded protein as they exist naturally. The disadvantage is that only small proteins lend themselves to this type of experiment. We compute the sample mean and variance of the *C_α_* coordinates over the different models and assume that each *C_α_* coordinate is normally distributed as:

$$\begin{array}{lll} x_{i}\sim N({\bar{x}}_{i},s^{2}\sigma_{x}), & y_{i}\sim N({\bar{y}}_{i},s^{2}\sigma_{y}), & \begin{array}{ll} \text{and} & z_{i}\sim N({\bar{z}}_{i},s^{2}\sigma_{z}), \end{array} \end{array}$$

where the means are sample means and the variances are scaled sample variances. The use of the scale parameter is discussed momentarily.

X-ray crystallography requires that a protein first be crystallized, and a solution must become saturated enough to create a crystalline structure that can then be imaged with X-rays. The advantage over NMR is that X-ray crystallography more easily accommodates large proteins. The disadvantage is that the 3D descriptions are not of the proteins in a natural aqueous solution. Uncertainty for X-ray crystallography experiments is expressed by a list of B-factors, each of which assesses the variation of the spacial coordinates of a specific atom. A B-factor, often called a Debye-Waller factor, is a scaled version of the mean squared displacement of an atom. If *r̄_i_* is the mean of the *i*-th residue's *C_α_* coordinates over the crystalline structure and *r_i_* is the random position of the atom, then the B-factor of the *i*-th residue is:

$$B_{i}=8\pi^{2}\;\text{E}\left({\left(r_{i}-{\bar{r}}_{i}\right)}^{T}\left(r_{i}-{\bar{r}}_{i}\right)\right)=8\pi^{2}\sigma_{i}^{2},$$

where E is the expected value and *σ^2^* is the variance. To assess EIGAs' sensitivity to coordinate perturbation, we assume that the coordinates of the *C_α_* atoms are uncorrelated normals, whose variances are scalar multiples of a third of $\sigma_{i}^{2}$. Hence, for each X-ray crystallography experiment, the coordinates of the *C_α_* atoms are the random variables:

(2) $$\begin{array}{lll} x_{i}\sim N\;\left({\bar{x}}_{i},\frac{s^{2}B_{i}}{24\pi^{2}}\right), & y_{i}\sim N\;\left({\bar{y}}_{i},\frac{s^{2}B_{i}}{24\pi^{2}}\right), & \begin{array}{ll} \text{and} & z_{i}\sim N\;\left({\bar{z}}_{i},\frac{s^{2}B_{i}}{24\pi^{2}}\right), \end{array} \end{array}$$

where *s* scales the standard deviation.

The reason for scaling the variances is that it provides a tool to adjust coordinate uncertainty, and hence, it can be used to assess an algorithm's sensitivity to coordinate uncertainty. A robust algorithm will remain effective for large values of *s*, whereas a less robust algorithm's efficacy will degrade for small values of *s*. For example, algorithms that depend on secondary structure assignments may be unduly sensitive to small coordinate variations. If an algorithm remains stable for *s* up to one, then the algorithm is robust with regards to the coordinate variations of the experiment.

Before continuing, we address an important criticism of using uncorrelated distributions for coordinate perturbations. Atomic coordinates are not uncorrelated, and for this reason, the uncorrelated assumption will over estimate coordinate variability. However, should an algorithm remain stable for large *s*, then we have some confidence that it will remain stable over the more limited perturbations that are actually possible. Hence, the test used here to assess experimental robustness is one-sided in that an algorithm may exhibit a loss of efficacy for small *s* but remain accurate over the range of actual variation. With only a B-factor available, the uncorrelated assumption leads to a reasonable test that is independent of a full simulation of each protein's dynamics. Such simulations are computationally costly and would negate our ability to harness the alignment efficiency of EIGAs to consider experimental robustness.

To illustrate the effect of randomizing the coordinates, we consider 1QMPa with each coordinate being distributed as in (2). An image of the static protein in terms of secondary structure is shown in Figure 5 on the left (images generated by PyMOL [44]). A random sample of the same protein with its coordinates being drawn from the normal distributions with *s* = 1 is depicted in the center. The difference in the two illustrates that secondary structures can be lost as the coordinates vary. We mention that after looking at several such comparisons, the atom (sphere) view commonly shows that the random perturbations maintain the general globular shape, even though secondary structures are lost. This observation suggests that an alignment algorithm that directly compares backbone atoms might be more stable with regards to coordinate uncertainty than an algorithm that depends on secondary structure.

**Figure 6:**
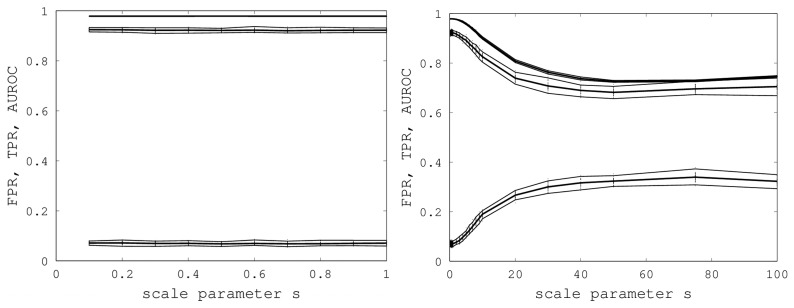
On the left, AUROC (top) and the best TPR (middle) and FPR (bottom) are shown as *s* ranges from 0.1 to one. On the right, AUROC (top) and the best TPR (middle) and FPR (bottom) as *s* ranges from 0.1 to 100. 95% bootstrap confidence intervals are shown in both graphs for all simulations.

We initially varied *s* from zero to one in steps of 0.1, and for each *s*, we generated 40 random samples of the Proteus300 dataset [45]. The reason for 40 samples was that this nicely yielded a 95% bootstrap confidence interval by removing the highest and lowest AUROC. We found that EIGAs' efficacy was essentially unaffected for this range of *s*. A graph of AUROC and the best TPR and FPR is shown on the left in Figure 6. Over all 400 samples, as *s* increased from 0.1 to one, the AUROC was always above 0.9783 and below 0.9785 and, hence, was nearly constant. The best TPR (FPR) was between 0.9201 (0.0678) and 0.9244 (0.0715). We conclude that EIGAs is robust with respect to coordinate uncertainty.

The success of EIGAs for *s* ≤ 1 led to the question of how much coordinate variability EIGAs could accommodate before AUROC degraded significantly. To answer this question, we let *s* range over {1, 2, …, 10, 20, 30, 40, 50, 75, 100}, again using 40 samples for each *s*. The results are shown on the right in Figure 6. AUROC and the best TPR and FPR degraded as *s* increased from one to 20, but then, these metrics flattened as *s* increased to 100. Indeed, from 50 to 100, AUROC is nearly constant at 0.7332, and the best TPR and FPR are nearly constant at 0.6941 and 0.3284. This is a surprising result, as it shows that EIGAs can correctly match proteins within a family with about 70% accuracy, even with a 100-fold increase in coordinate variation.

## 7. Conclusions and Future Research

The immediate conclusion of our numerical work is that EIGAs is robust against variations that could alter the DP algorithm. Moreover, our adapted three-fold cross-validation suggests that EIGAs can be tuned on moderate-sized datasets and then applied to larger collections. A secondary conclusion is that the modern DP-based algorithms are likely to exhibit similar robustness, since DP can be stable over wide ranges of input data.

The results for large values of *s* with regard to coordinate uncertainty are not what we had expected. The B-factors are not uniform across the atoms, and we suspect that the smaller B-factors correlate with the hydrophobic core, which is likely (much) more stable. The fact that EIGAs remains effective for large values of *s* suggests that it is aligning structures based on their most stable elements.

A near term research question is to test several of the other DP algorithms designed for fast alignments to verify whether or not they share EIGAs' robustness. If so, then a logical conclusion would be that DP is the reason why efficient algorithms are finding success for a variety of different similarity models.
